# Legal Lens on Hysteroscopy: A Retrospective Review of Medical Malpractice Claims of Hysteroscopic Procedures

**DOI:** 10.3390/healthcare13030264

**Published:** 2025-01-29

**Authors:** Adriana C. Baez, Staci Marbin, Jose Carugno

**Affiliations:** 1 Miller School of Medicine, University of Miami, Miami, FL 33146, USA; adrianabaez@med.miami.edu; 2 Obstetrics and Gynecology Department, Miller School of Medicine, University of Miami, Miami, FL 33146, USA; sjm53@miami.edu

**Keywords:** hysteroscopy, litigation, malpractice

## Abstract

**Background**: Given the expansiveness of diagnostic and therapeutic hysteroscopy, promptly recognizing complications and intervening as necessary to prevent adverse outcomes and minimize legal risk is imperative. We aim to describe the litigious trends in hysteroscopic procedures across the United States; **Methods**: Publicly available lawsuits published on a well-known legal database, Westlaw, were reviewed. The search term “’hysteroscop!’” was used to filter cases for court opinions containing words with this prefix, including “hysteroscopy”, “hysteroscopies”, and “hysteroscopic.” Adverse events, procedural outcomes, post-procedural surgery urgency, and resultant disabilities were extracted for themes; **Results**: The primary complication resulting in legal action was uterine perforation (52.9%), followed by claimed technical mistakes (23.5%) and damage to surrounding structures (11.8%). The most common outcome was the need for future corrective surgery (70.6%), and a number of cases resulted in permanent brain damage or death (23.6%). Of the patients that required subsequent corrective surgery (n = 12), 91.7% of them required emergent surgery. Patients sought damages most commonly from individual attending providers (41.2%), attending physician and practice groups/hospitals collectively (41.2%), and additional entities, including resident physicians in training and manufacturers. Awards ranged from $322,308 to $9,387,109; **Conclusions**: Medical malpractice litigation is challenging to patients and providers alike, often leading to financial, emotional, and professional burden. Our evaluation highlights the variability in adverse events from hysteroscopy which prompt litigation, financial burden of lawsuits, and legal risk of individual providers, in an attempt to improve safety of future procedures.

## 1. Introduction

Hysteroscopy is currently considered the gold standard for the evaluation of intrauterine pathology [1,2]. While hysteroscopy has traditionally been performed in the operating room setting, the miniaturization of the hysteroscope and the recognition of safety has resulted in more procedures being performed in the office setting [1,3]. Studies support the general safety of the procedure, including in the evaluation of complex oncologic pathology pre-operatively [4] and in women with certain comorbidities [5]. The trend of hysteroscopic procedures has grown from 197,800 outpatient hysteroscopies in 1994, to 225,900 in 1995, and then 232,000 in 1996 [6]. The market size for hysteroscopy was estimated at $4.5 billion in 2023 and is expected to grow at a compound annual growth rate of 7.5% in the next 15 years [7]. Although hysteroscopy is being aggressively incorporated in gynecologic clinical practice in the United States over the last few years, in 2018, only around 25% of gynecologists reported performing in-office hysteroscopy [8].

In-office hysteroscopy confers multiple significant advantages for both patients and healthcare providers. This modality enables patients to promptly resume their daily activities post-procedure, thereby enhancing patient convenience and satisfaction [9]. By circumventing the need for anesthesia and the operating room, this approach mitigates associated risks and costs [10,11]. Moreover, the “see and treat” strategy allows for the immediate diagnosis and management of various intrauterine pathologies in a single visit [12,13]. In a questionnaire given to 240 women, most women reported satisfaction with this approach, noting only mild or moderate pain, resolution of pain in under 20 min, and an inclination for this choice of treatment if given the option in the future [14]. As a result, in-office hysteroscopy not only improves patient outcomes but also optimizes healthcare efficiency and resource allocation, thus benefiting the broader medical practice.

Reasons for hesitancy in offering this procedure in the office setting include inadequate physician training, concerns about patient discomfort, and the initial cost of equipment, all which factor into a physician’s decision to offer this type of procedure [15]. An analysis of resident comfort with in-office hysteroscopy revealed that only 26% of residents received training, and that only 14% of senior residents expressed comfort performing this procedure in the office setting [16]. Regardless of the setting, the procedure itself is associated with certain risks and complications. Aside from the complications common to all surgical procedures—damage to surrounding tissues, excessive blood loss, infection, and anesthetic complications—complications can arise with the use of distention media or electrosurgical instruments [17]. These include fluid overload, uterine perforation, air embolism, and thermal injury [17]. Full disclosure of these complications during the consent process is imperative.

Unique to the field of obstetrics and gynecology (OBGYN) is the higher rate of malpractice lawsuits compared to most other specialties, where, in 2021, obstetrician-gynecologists (OBGYNs) were the fifth most likely specialists to be sued for malpractice [18]. In the 2021 Medscape Ob/Gyn Malpractice Report, 79% of OBGYNs in the United States reported having been named as a defendant in at least one medical malpractice lawsuit [18]. A 2012 study analyzing malpractice data acquired from a medical professional liability insurer of over 40,000 doctors projected that, by age 45, 74% of OBGYNS were projected to face a medical malpractice claim [19]. Further, in a study of 46 published medical malpractice cases in OBGYN, residents, fellows, and even medical students have been named as defendants at alarmingly high rates (95.6%, 4.4%, and 13.0%, respectively) [20]. The comparatively higher risk of litigation is not unique to the United States [21,22]. Not only is the likelihood of finding oneself the target of litigation higher than most fields, but, at 18%, OBGYNs also see the highest proportion of catastrophic damage payments (>$1 million) [23]. Others calculate they are second in this category only to neurosurgeons [24]. As such, it is imperative for current and future OBGYNs to understand their legal risk.

Analyzing the litigatory landscape of hysteroscopy provides multiple benefits. Examination of legal claims sheds light on the nature and severity of patient outcomes, enabling healthcare providers to mitigate similar adverse experiences in their own patient populations. Implementing targeted risk-avoidance strategies enhances patient outcomes for future hysteroscopy procedures and fosters greater confidence among providers, thereby potentially expanding the use of this minimally invasive technique to a greater body of patients.

## 2. Materials and Methods

### 2.1. Study Design and Data Source

In this retrospective, narrative review, we queried the publicly available data on the Thomson Reuters Westlaw Database (Westlaw), which is a legal database containing published United States lawsuits and secondary sources. Lawsuits published in this database include trial court, appellate, and supreme court cases across state and federal jurisdictions. While the database contains tens of thousands of cases, there are no federal or state mandates dictating criteria for case law publication. As such, the cases acquired are not an exhaustive list of all cases that have been brought or heard. Such a search would be presently impossible, given that no legal database publishes every single case, nor do the various court reporters. Similarly, there are no criteria for the format or level of detail published in each opinion, and as such, the explanation of events for each case varies. In this study, we used the term “hysteroscop!” to filter cases for any court opinion containing words with this prefix, including “hysteroscopy”, “hysteroscopies”, and “hysteroscopic” (N = 258).

### 2.2. Case Selection

The database was searched for cases published since the database’s inception in 1975 through 1 January 2024. Exclusion criteria included adverse events that were related, yet not directly occurring as a result of the hysteroscopic procedure. Lawsuits against defendants for failure to pursue hysteroscopy as a means of diagnostic or therapeutic procedure, according to the standard of care, were also excluded, as these cases were not directly related to the hysteroscopic procedure. The authors reviewed the full opinion in each case for relevancy and excluded all cases unrelated to hysteroscopy (228 cases, 88.4%). Thirteen of the remaining 30 cases were removed due to harm not directly related to the hysteroscopic procedure. A total of 17 cases in which the adverse event was directly related to a hysteroscopic procedure were included in the final analysis. (See Table 1). The remaining 13 cases not used in the analysis can be found in Appendix A, Table A1.

### 2.3. Data Abstraction and Analysis

**Figure 1:**
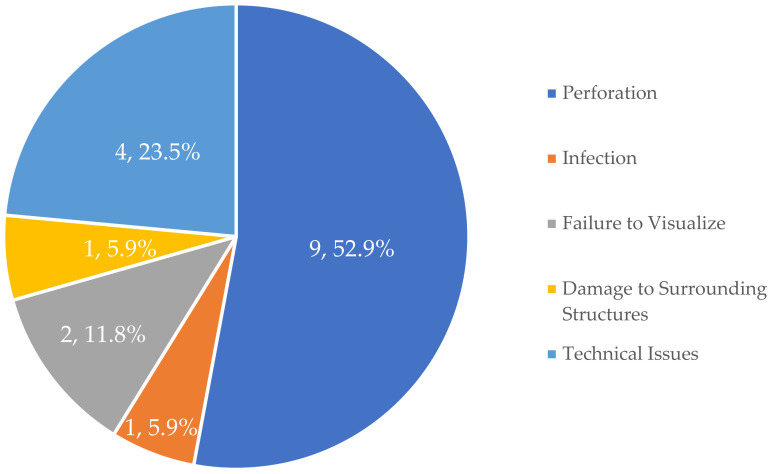
Adverse events occurring during hysteroscopy.

The authors reviewed the full opinions for each case and abstracted variables, including procedural data, such as court decision, complaint, parties, and holding—and medical outcome data, such as adverse event, health outcome, and timing of adverse event in relation to the procedure. Data were then analyzed with IBM SPSS Statistics Version 28.0.2.0. As cases often allege multiple complaints, only the primary complaint was extracted for analysis. Figure 1 reviews all 17 cases and their primary harm.

## 3. Results

Resultant cases were decided between 1999 and 2023. Cases were distributed across 12 States, with California (n = 3, 17.6%) and Louisiana (n = 3, 17.6%) being equally common, followed by New Jersey (n = 2, 11.8%). The additional states included Georgia, Texas, Massachusetts, New York, Washington DC, Mississippi, Kansas, New Mexico, and Nevada. The judge found most often for the plaintiff (n = 6, 35.3%), followed by summary judgment in favor of the defendant (n = 3, 17.6%). Two cases (11.8%) were dismissed. Notably, final holdings were unable to be assessed in those that were unpublished (n = 2, 11.8%) or reversed and remanded. For example, a trial court may grant summary judgment dismissing a case, but the appellate court may reverse the holding and remand it to the trial court to be heard again. Without further published opinions, it is impossible to determine for which party the outcome was favorable.

The vast majority of cases alleged negligence on behalf of the defendant(s) (n = 20, 95.2%), the four elements of negligence being (1) duty of care, (2) breach of the existing duty, (3) direct causation of harm as a result of the breach, and (4) damages sought for actual harm [25]. The remaining case was brought for lack of informed consent. The defendants in these cases were largely individual gynecologist specialists (n = 7, 41.2%) and both gynecology specialists and their affiliated hospital or representative company (n = 7, 41.2%). Two cases cited manufacturers as additional defendants, and one included a resident physician in training.

Most cases resulted in disability or harm requiring further intervention (n = 15, 88.2%), with two cases resulting in death. However, of those suffering harm or disability, almost all of the cases requiring corrective surgery (n = 12) were emergent (n = 11, 91.7%). The remaining cases allegedly experienced future pain and suffering (n = 1, 5.9%) or permanent brain damage (n = 2, 11.8%). Corrective surgery ranged from the repair of perforated organs (most often the uterus and/or bowel) to total hysterectomy, resulting in infertility. Figure 1 depicts the types of adverse event experiences by the plaintiffs in these cases.

Damages were awarded in six (35.3%) of the published opinions and were unknown in three cases, whether due to lack of inclusion in the published opinion or remand to a lower court. With damages totaling between $322,308 and $9,387,109, the most expensive lawsuit was for the improper operation of the fluid management system, resulting in a persistent vegetative state of the patient. In fact, two of the most expensive cases were brought for permanent peripheral or central nervous system damage following the misuse of common hysteroscopic appliances.

## 4. Discussion

The analysis of medical malpractice claims related to hysteroscopy offers critical insights into the litigious trends and potential risk factors associated with this commonly performed gynecologic procedure. While a minority of cases resulted in monetary compensation, the cost of litigation of medical malpractice cases can be expensive for defendants, regardless of the outcome. One law firm estimates the initial cost of an expert witness opinion at $10,000 in addition to the estimated $20,000–$50,000 cost of preparing and filing a case [26]. This sum is derived, in part, by attorney’s fees (often $300–$500 per hour) and filing fees ($0.25–$2 per page) [27]. Aside from the economic burden, medical malpractice cases can take several months to years to be resolved. During some of this time, the physician will naturally be required to take time away from their clinical duties. In addition, the emotional burden of being a defendant in a medical malpractice can be taxing. One survey reported that 25% of doctors who were sued characterized the experience as “Horrible; one of the worst experiences of my life”, and more than 60% described it as “Very bad; disruptive and humiliating” [28].

In contrast with other medical malpractice reviews, defendants in the cases in this study were more likely to be individual gynecology specialists or group practices, and one case involved a resident in training. This may be due to a relatively higher technicality of hysteroscopic procedures, which are more likely to involve more experienced physicians. Because hysteroscopy is a minimally invasive “single-operator” procedure requiring very little assistance, gynecologists with the requisite training and experience may perform this procedure with basic staff support.

Uterine perforation was the most cited harm in the complaint. Most uterine perforations may be underreported, as many go unnoticed during the procedure or do not require further surgical interventions. Notably, diagnostic is less invasive than operative hysteroscopy and thus carries a lower risk of perforation. Of course, vital signs and changes in clinical status should be monitored after the procedure. The claims reported in this study caused damage to surrounding tissues, such as the bowel, leaving the body susceptible to infection and the need for further surgical interventions. Additional structural damage led to infertility. At a time when some women may be of child-bearing age or trying to conceive, this is obviously a devastating outcome.

As hysteroscopy is intended to be a minimally invasive procedure, visualization is limited compared to laparoscopic surgery. Comfort with procedural tools, including the hysteroscope and fluid management systems, is mandatory to circumvent unintentional oversight. Moreover, clinician and support staff expertise with the procedure, emergency planning, and proper equipment inventory are absolutely imperative before deciding to utilize the outpatient hysteroscopic approach.

Other cases cited improper use of tools not related to visualization, for example, the case in which the patient died after absorbing 11 L of glycine during her surgical procedure. The advanced tools and techniques in hysteroscopy require the comfort of the presiding physician and the rest of the procedural team. Coordination, communication, and feedback are important tools to utilize across the team, in all procedures. Of the potential risks communicated to patients during the informed consent process, these harms are examples of sentinel events that systemic safeguards are designed to prevent. While the role is shared by the entire procedural team, the surgeon is ultimately responsible for verifying patient information, applicable consents, and procedural plan before, during, and after the procedure.

The decision to perform a hysteroscopy in the outpatient versus inpatient setting should consider a variety of factors, including comorbidities and availability of equipment. While in-office hysteroscopy is more convenient for both the patient and the physician, the lack of anesthesiologists and limited resuscitation technology may dissuade certain gynecologists from opting for this technique in the office setting. Complex patients are often scheduled for OR-based hysteroscopy. Currently, no guidelines exist detailing which patients are better suited for in-office versus OR-based procedures, and more research is warranted to assist physicians in making this important decision. Regardless, proper procedural and emergency equipment are necessities.

Nine cases in the search detailed harm not directly related to a single hysteroscopic procedure but are included in this discussion for informational purposes (See Appendix A, Table A1). Several cases were brought for failure to perform hysteroscopy in a timely manner, which were considered a deviation from the standard of care. Without knowing exactly what the clinical reasoning or barriers were in these cases, it can still be emphasized that the use of in-office hysteroscopy may assist in circumventing barriers to OR-based hysteroscopy (scheduling, cost, availability, etc.). Current research, albeit limited, suggests that physician inexperience may contribute to hesitancy to perform office-based hysteroscopy [29,30]. Further research should be performed to understand the deterrents to performing this procedure.

A few limitations of the study should be noted; most importantly, not all lawsuits are published within the Westlaw or any other legal database. This includes the sequelae to currently published cases. Cases are not published according to certain criteria, but rather by the subjective importance that the opinion carries. Though the true number of medical malpractice cases related to hysteroscopy is unknown, it can be assumed that our study underestimates the number of severe surgical complications during hysteroscopy that occurred during the study period. Consequently, it may not fully represent additional potential adverse events that have not been addressed in our review. Comparably minor adverse events or a higher volume of minor adverse events, similarly, may not be published.

Additionally, the outcomes of each published case depend on several procedural factors, including standing, statute of limitations, and failure to state a complaint. In this way, specific final outcomes detailing who the prevailing party was are not available either due to a lack of publishing or continuation of the case. Thus, the outcomes presented in this study should be interpreted in the setting of the legal, procedural, and data availability factors which are unique to each case and the complexity of civil procedure.

## 5. Conclusions

Hysteroscopic procedures, although safe, can lead to adverse events that generate medical malpractice litigation. Safe clinical practice within the boundaries of the standard of care aids in mitigating a physician’s legal risk. Doing so may also protect against the emotional and financial toll of litigation on physicians and patients. Physicians performing hysteroscopies should always uphold the principle of the Hippocratic Oath, “*primum non nocere*” (first, do no harm), to minimize risks and protect patient well-being.

## Figures and Tables

**Table 1:** Cases directly related to hysteroscopy.

Year of Case Decision	Jurisdiction	Adverse Event	Outcome	Holding
2004	Supreme Court, Appellate Division, Third Department, New York (NY)	Patient absorbed 11 L of glycine during her surgery and died	Death	Affirmed
2013	Court of Appeals of Georgia (GA)	Uterine wall perforation, small bowel perforation	Harm	No final decision
1999	Supreme Court of New Jersey (NJ)	Massive air embolism from incorrect tube connection to the hysteroscope, causing a closed-circuit pathway that permitted the nitrogen gas to enter the patient’s uterus and resulted in the embolism	Death	Plaintiff, $2,000,000
2005	Court of Appeal, Fourth District, Division 3, California (CA)	Negligent combination of the wrong pump (pressure) and the wrong fluid meant sucking large volumes of water into Hamels vascular system, causing disintegration of blood cells, electrolyte imbalance, and ultimately a massive pulmonary edema and cardiac arrest	Disability	Plaintiff, $9,387,109
2000	Court of Appeals of Texas, Houston, 1st District (TX)	Failing to discover the patient had an intra-uterine device (IUD) in her uterus during hysteroscopy and while she was being treated for infertility	Harm	Summary Judgment (for Defendant)
2000	Court of Appeal of Louisiana (LA)	Failure to cool metal instruments resulting in third degree burns, requiring surgical debridement and, subsequently, skin graft	Harm	Plaintiff, $395,000
2017	United States District Court, D. New Jersey (NJ)	Bowel and aortic injury	Harm	Summary judgment (for Defendant)
2009	Court of Appeal of Louisiana (LA)	Failure to diagnose and repair damage to uterine artery resulting in hysterectomy	Harm	Dismissed
2017	Court of Appeals of Kansas (KS)	Uterus perforation, causing heated thermal ablation fluid to spill into the abdominal cavity, severely injuring her bowel	Harm	Plaintiff, $322,308
2001	Superior Court of Massachusetts (MA)	Perforated uterine wall, severed uterine artery, and severed nerve	Disability	No final decision (unpublished)
2015	Supreme Court of Mississippi (MS)	Uterine perforation and small bowel burn injuries allegedly sustained during an endometrial ablation procedure	Harm	Dismissed against one party; remanded for remaining
2016	Court of Appeal of Louisiana, Fourth Circuit (LA)	Administration of a known allergen to patient prior to in-office hysteroscopy and the presence of multiple comorbidities, leading to respiratory failure, cardiac arrest, and permanent brain damage	Disability	Plaintiff, $627,281.72
2010	District of Columbia Court of Appeals (DC)	Uterine and bowel perforation	Harm	Defendant
2021	Supreme Court of New Mexico (NM)	Uterine and bowel perforation	Harm	Plaintiff, $2,600,000
2023	Supreme Court of Nevada (NV)	Uterine and bowel perforation	Harm	Reversed and remanded
2013	Court of Appeal, Fourth District, Division 1, California (CA)	Uterine and bowel perforation	Harm	Summary Judgment
2019	Court of Appeal, Sixth District, California (CA)	VP shunt infection after hysteroscopy performed without antibiotics	Harm	Defendant

## Data Availability

No new data were created or analyzed in this study. Data sharing is not applicable to this article.

## Appendix A

**Table A1:** Cases indirectly related to hysteroscopy (not included in the analysis).

Year	Jurisdiction	Adverse Event	Outcome	Holding
2017	Court of Appeals of Tennessee (TN)	Multiple failed hysteroscopies resulting in several adhesions, which led to hysterectomy with severe complications (ICU transfer, enterectomy with anastomosis to resolve through and through bowel perforation)	Harm	Defendant
2010	Supreme Court of Connecticut (CT)	Physician misread hysteroscopic D&C pathology report of benign condition and performed unnecessary hysterectomy and bilateral salpingooopherectomy; failed to inform patient of benign condition	Disability	Plaintiff $5,200,000
2012	Superior Court of Delaware (DE)	Failure to perform timely hysterectomy, delaying diagnosis of endometrial cancer	Harm	Summary Judgment
2012	United States District Court, District of Columbia (DC)	Failure to perform timely hysterectomy. An earlier procedure would have diagnosed her condition as pre-malignancy or early-stage, rather than Stage IVA cervical cancer, as her doctors ultimately diagnosed	Harm	Defendant
1996	Court of Appeal of Louisiana (LA)	TMJ dislocation during intubation	Harm	Plaintiff $379,672
2001	Supreme Court, Nassau County, New York (NY)	Failing to perform timely hysteroscopy to treat placenta retention following childbirth, resulting in acute inflammation and infection. Subsequent adhesions led to multiple miscarriages and failed corrective surgeries	Harm	Partial Summary Judgment
2016	Supreme Court, Appellate Division, Second Department, New York (NY)	Wrongful death based on allegations that oncologist failed to timely perform hysteroscopy to diagnose decedent’s fallopian tube cancer, which resulted in spread of disease and decedent’s eventual death	Death	Summary Judgment denied will go to trial
2005	Court of Appeals of Texas, Waco (TX)	Failure to perform proper diagnostic work up with hysteroscopy, instead performing hysterectomy where permanent injuries ensued	Disability	Dismissed (in favor of defendant)
2008	Supreme Court, New York County, New York (NY)	Failure to perform proper diagnostic work up with hysteroscopy, which may have led to early diagnosis of cancer	Death	Summary Judgment denied will go to trial
2019	Court of Appeals of Michigan (MI)	Permanent damage to fallopian tubes	Disability	Summary Judgment (for Defendant)
1997	Supreme Court of Georgia (GA)	Negligent use of a laser device resulting in permanent brain damage	Disability	Reversed and remanded
1997	Appellate Court of Illinois (IL)	Tubal ligation without consent	Harm	Reversed and remanded
1993	United States Court of Appeals, Second Circuit (VT)	Negligent use of a retractor, entrapping lateral femoral cutaneous nerve and causing permanent loss of function of the leg.	Disability	Plaintiff, $800,000
